# Development of MRI-based radiomics predictive model for classifying endometrial lesions

**DOI:** 10.1038/s41598-023-28819-2

**Published:** 2023-01-28

**Authors:** Jiaqi Liu, Shiyun Li, Huashan Lin, Peiei Pang, Puying Luo, Bing Fan, Juhong Yu

**Affiliations:** 1 Department of Radiology, Jiangxi Provincial People’s Hospital, The First Affiliated Hospital of Nanchang Medical College, 152 Aiguo Road, Nanchang, 330006 China; 2 Department of Gynecology, Jiangxi Provincial People’s Hospital, The First Affiliated Hospital of Nanchang Medical College, Nanchang, China; 3 GE Healthcare, Hangzhou, China

**Keywords:** Cancer, Diseases, Medical research, Oncology

## Abstract

An unbiased and accurate diagnosis of benign and malignant endometrial lesions is essential for the gynecologist, as each type might require distinct treatment. Radiomics is a quantitative method that could facilitate deep mining of information and quantification of the heterogeneity in images, thereby aiding clinicians in proper lesion diagnosis. The aim of this study is to develop an appropriate predictive model for the classification of benign and malignant endometrial lesions, and evaluate potential clinical applicability of the model. 139 patients with pathologically-confirmed endometrial lesions from January 2018 to July 2020 in two independent centers (center A and B) were finally analyzed. Center A was used for training set, while center B was used for test set. The lesions were manually drawn on the largest slice based on the lesion area by two radiologists. After feature extraction and feature selection, the possible associations between radiomics features and clinical parameters were assessed by Uni- and multi- variable logistic regression. The receiver operator characteristic (ROC) curve and DeLong validation were employed to evaluate the possible predictive performance of the models. Decision curve analysis (DCA) was used to evaluate the net benefit of the radiomics nomogram. A radiomics prediction model was established from the 15 selected features, and were found to be relatively high discriminative on the basis of the area under the ROC curve (AUC) for both the training and the test cohorts (AUC = 0.90 and 0.85, respectively). The radiomics nomogram also showed good performance of discrimination for both the training and test cohorts (AUC = 0.91 and 0.86, respectively), and the DeLong test shows that AUCs were significantly different between clinical parameters and nomogram. The result of DCA demonstrated the clinical usefulness of this novel nomogram method. The predictive model constructed based on MRI radiomics and clinical parameters indicated a highly diagnostic efficiency, thereby implying its potential clinical usefulness for the precise identification and prediction of endometrial lesions.

## Introduction

Endometrial lesions are commonly occurring diseases of the female reproductive system which can cause infertility or lead to an abnormal uterine bleeding^1,2^. As a consequence of the ageing population and increasing obesity rate^3^, the incidence of endometrial cancer (EC) is rapidly rising and it has become one of the most commonly diagnosed gynaecological malignancy in the developed world^4^. As a result of the tremendous advantage of significantly higher resolution, magnetic resonance imaging (MRI) could illustrate the endometrial conditions clearly, as well as the form the basis of imaging option for newly diagnosed EC patients^5^. However, an accurate and objective assessment of abnormalities related to the endometrium could pose a range of challenges to the radiologists and gynecologists. On the one hand, the normal endometrium is a dynamic tissue, that can be predominantly influenced by the age, menopausal status, menstrual cycle and the hormonal therapy^6^. Moreover, it has been found that overlapping image characters and clinical features of benign and malignant lesions might be detected simultaneously^1,7^. The varying sizes of the endometrial lesions pose a significant challenge to accurate diagnosis; presence of large lesions can lead to an abnormal appearance of endometrium and confuse exact origin of histology while small lesions that frequently overlap with the normal tissue are often ignored^8^. Transvaginal ultrasound and hysteroscopy have also been found to be helpful for the diagnosis of endometrial lesions, but both are dependent on the experience of the surgeons involved^8,9^, leading to a relatively high subjectivity and variability. Therefore, an accurate and precise differentiation between the benign and malignant endometrial lesions is important for efficient treatment.

Radiomics, a rapidly developing science, which can effectively convert the digital images into mineable high-dimensional data and thus clearly reflect biomedical information underlying both pathophysiological conditions and tumor heterogeneity^10,11^. It has been widely used to monitor the progression of the various diseases because it can overcome the deficiency of image interpretation by the human visual perception and thereby result in more objective and accurate information^10^. Our group has previously used CT-based radiomics to develop an automated diagnosis approach for the detection of ovarian neoplasm malignancy and obtained satisfactory results^12^. There are several reports that have focused on the precise evaluation of preoperative assessment of EC^13–15^. However, a reliable primary differential diagnosis of endometrial lesions is also urgently needed to guide the gynecologist to select an appropriate treatment. In this study, we hypothesized that imaging biomarkers could be potentially developed non-invasively to facilitate the differentiation between the benign and malignant diseases on the basis of MRI-based radiomics data that was extracted from the primary endometrial lesions.

## Materials and methods

### Study participants

**Figure 1:**
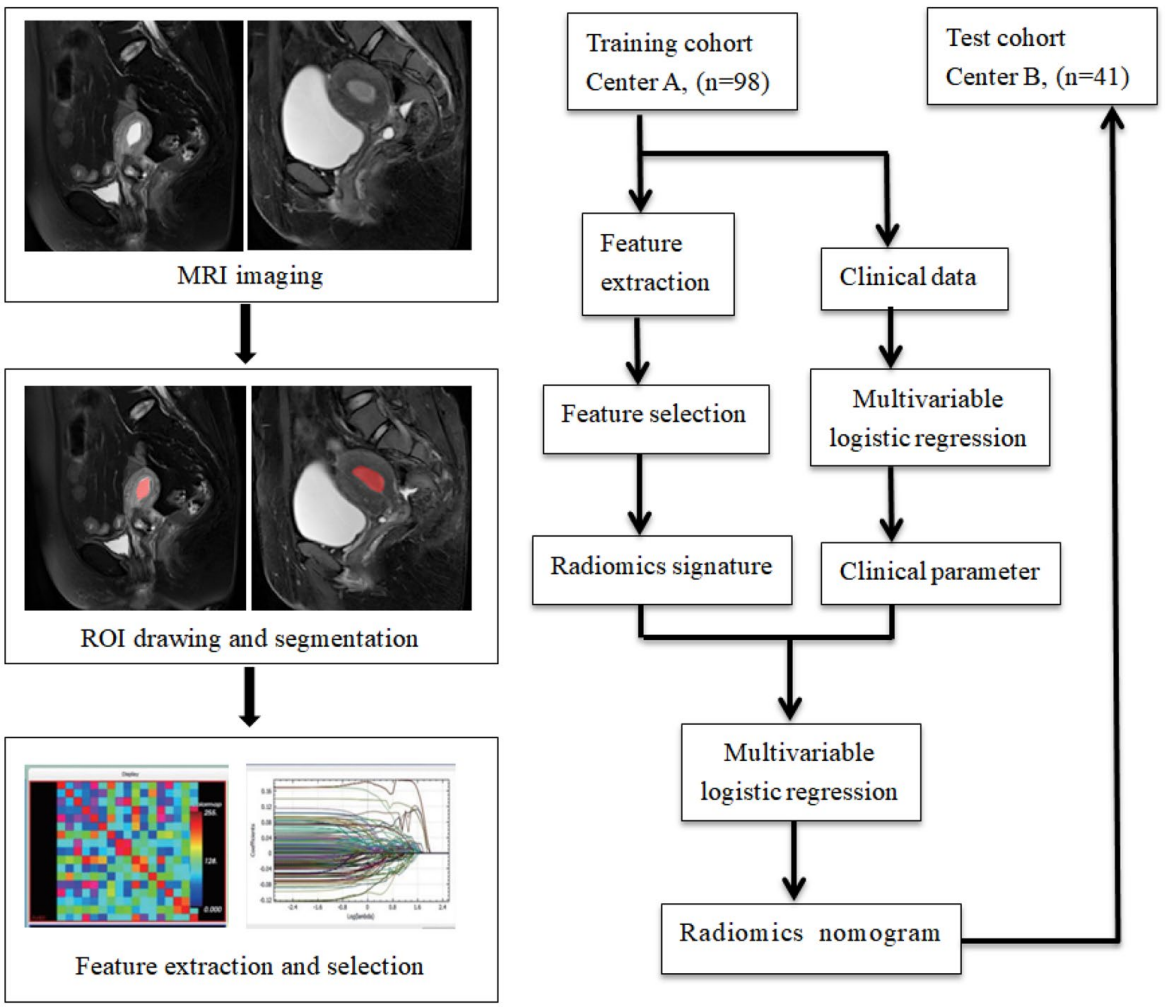
A diagram depicting overview of the study’s workflow.

This retrospective study was performed by two independent centers jointly (center A and B). During January 2018 to July 2020, 164 consecutive histologically diagnosed endometrial lesions patients with preoperative MRI were independently collected at center A and B. These endometrial lesions encompassed benign and malignant endometrial diseases, including EC, endometrial hyperplasia, submucous myomas, endometrial polyps and endometritis. Inclusion criteria used were as follows: (1) female patients with histopathologically verified endometrial lesions, (2) patients without previous or current history of malignancy other than endometrial tumors, (3) no preoperative systemic therapy, (4) MRI performed within 30 days before the gynecological surgery. Exclusion criteria used were as follows: (1) low-quality imaging records (n = 3), (2) without surgical information (n = 12), (3) preoperative systemic therapy (n = 3), (4) malignancy other than EC (n = 2), (5) accompanied with distinct benign and malignancy lesions of endometrium (n = 5). Finally, a total of 139 patients were enrolled in this study, including 98 patients (with 46 and 52 patients with benign and malignant endometrial lesions respectively) treated in center A were assigned to the training cohort, whereas the 41 patients (with 19 and 22 patients corresponding to benign and malignant endometrial lesion respectively) treated in center B were assigned to the test cohort. An overview of the study’s workflow has been illustrated in Fig. 1.

### MR imaging

The MR images were obtained by using the 3 T Trio Siemens scanner (center A) and 3 T Magnetom Skyra Siemens scanner (center B) with phased-array abdominal coils. All patients were required to breathe freely in supine position during the data acquisition. The following sequences were obtained: axial T1-weighed imaging (T1WI), axial T2-weighed imaging (T2WI), coronal and sagittal T2-weighed imaging with fat saturation (FS T2WI), and diffusion weighed imaging (DWI) with a b value of 0 and 800 s/mm2 with an apparent dispersion coefficient (ADC) map. FS T2WI images were acquired using turbo spin echo with fat saturation (repetition time (TR)/echo time (TE) = 3960/84, matrix = 512 × 512, filed of view (FOV) = 350 × 350 mm^2^, slice thickness = 4.0 mm, average = 1, voxel size = 0.8 × 0.8 × 4.0 mm^3^ for center A and TR/TE = 3200/101, matrix = 512 × 512, FOV = 207 × 207 mm^2^, slice thickness = 4.0 mm, average = 2, voxel size = 0.6 × 0.6 × 4.0 mm^3^ for center B).

### Imaging segmentation

All the regions of interest (ROI) were segmented using ITK-SNAP software (version 3.8.0, www.itksnap.org) starting from the baseline DICOM image. The manual ROI segmentation was performed from the largest lesion diameter slice^16^ in sagittal FS T2WI sequence by a radiologist (Reader A, with 5 years’ experience in abdominal MRI) who was blinded to the histopathology data of the patients. A month later, another radiologist (Reader B, with 10 years’ experience in abdominal MRI) were randomly selected images obtained from 30 different and repeated the manual ROI drawings. The extracted features of the ROIs from two the different readers were calculated using the intra-class correlation coefficient (ICC).

### Feature extraction and selection

Spatial resampling was performed before feature extraction. The original DICOM data of sagittal FS T2WI and the paired 2D ROIs were resampled by 1 × 1 × 1 voxel. Radiomics features including Histogram, Form Factor, Haralick, Gray-level co-occurrence matrix (GLCM), Gray-level zone-size matrix (GLZSM) and Gray-level Run-length matrix (GLRLM) were thereafter calculated by AK software (Artificial Intelligence Kit V 3.0.0R, GE Healthcare). GLCM and GLRLM in four different directions (0°, 45°, 90°, 135°) and three offsets^1,4,7^ were calculated to describe the possible spatial distribution or patterns. Finally, each ROI resulted in extraction of 396 distinct features.

There were three steps used for the preprocessing of the data. Firstly, the outliers were replaced by the median of the same features. Secondly, z-score normalization is applied to eliminate the differences in the value scale of the features. Thirdly, patients treated in center A were assigned to the training set (n = 98), and the test set consisted of patients only from center B (n = 41). Center A and B are two different institutions with different patients data and MRI scanning equipment, which is better to evaluate the generalization of the models by using them as the training and test sets respectively.

Prior to the feature reduction, intraclass correlation coefficient (ICC) was calculated for each feature from Reader A and Reader B to remove the poorly reproducible and less robust features. Additionally, only image features with ICC > 0.75 were considered as qualified features, thereby indicating a high reproducibility and agreement^14^, and were reserved for subsequent calculation.

The maximum relevance minimum redundancy (mRMR) package in R software (Version 3.4.4) was employed to remove the redundant and irrelevant features preliminarily in the training dataset, and the 20 most significant features were preserved. The least absolute shrinkage and selection operator (LASSO)^17^ algorithm was used to effectively compress the coefficients of extracted features and cause the regression coefficients to become zero, by constructing a specific penalty function, so as to achieve an effective reduction in the additional features. In this process, the ten-fold cross-validation was then used to accurately calculate the optimal parameter λ, which was obtained at the minimum objective function value of LASSO regression model.

### Model construction and assessment

The final selected features were used to construct the radiomics model, under the optimal parameter λ of LASSO algorithm. The radiomics signature was obtained for the sum of the product of the final selected features and their corresponding coefficients weighed value, which was equivalent to radiomics score (radscore).

In the training cohort, univariate logistic regression was performed to potentially screen the various clinical parameters for endometrial lesions, including age^18^, body mass index (BMI)^19,20^, status of menopause^21^, as well as the conditions of hypertension and diabetes^22^. Multivariate logistic regression and stepwise regression were utilized to choose the significant indicator, to facilitate the development of a more comprehensive and efficacious nomogram model for the differentiation of the benign and malignant endometrial diseases. Moreover, the novel nomogram model which combined clinical parameters and Radscore was established, based on the multivariate logistic regression.

Receiver operating characteristics (ROC) curve was used to evaluate the discriminatory ability of models. Patients from two centers were thereafter classified into benign- or malignant-probability groups based on the cutoff values of ROC curve, and the nomogram method could precisely indicate the probability of occurrence of endometrial malignancy. Hosmer–Lemeshow test and calibration curves were used to assess performance of the model. Finally, the decision curve analysis (DCA) was also performed to validate the feasibility of the nomogram.

### Statistical analysis

The statistical analysis was conducted by using the SPSS 22.0 software and the R software. The count data between the samples was compared by χ^2^ test. The normality of data was verified by Kolmogorov–Smirnov test. All of the different parameters from the two datasets were compared statistically. The normally distributed data was analyzed by *t* test, and skewed distributed data was analyzed by Mann–Whitney *U* test. The predictive diagnostic efficiency of the models was also evaluated by the area under the ROC curve (AUC) and its 95% confidence interval (95% CI) to determine the specificity, sensitivity, and accuracy of the data.

### Ethics approval and consent to participate

This retrospective study based on the use of anonymous data was approved by the Ethic Committee of Jiangxi Provincial People’s Hospital, and the requirement for informed consent was waived. All procedures performed in studies involving human participants were in accordance with the ethical standards of the institutional (Jiangxi Provincial People’s Hospital) and/or national research committee and with the 1964 Helsinki declaration and its later amendments or comparable ethical standards.

## Results

### Patient clinical characteristics

Overall, with regard to the indicators of age, BMI, status of menopause, prevalence of hypertension and diabetes, there were no significant differences observed between the training and test cohorts, as shown in Table 1. However, there were statistical differences in the various clinical parameters (age, menopause) between the benign and malignant endometrial diseases for the training cohort (*p* < 0.05), but no significant clinical parameters for the test cohort (*p* > 0.05).

**Table 1:** The various characteristics of endometrial lesions in patients in the training and test cohorts.

Characteristics	Training cohort		*p* value	Test cohort		*p* value	*p* value^a^
	Benign (n = 46)	Malignant (n = 52)		Benign (n = 19)	Malignant (n = 22)		
Age							
< 50	26	12	0.002	11	8	0.287	0.620
≥ 50	20	40		8	14		
BMI							
< 24	21	19	0.478	15	13	0.305	0.836
≥ 24	25	33		4	9		
Menopause							
Yes	14	33	0.002	6	14	0.083	0.784
No	32	19		13	8		
Hypertension							
Yes	9	18	0.151	1	2	1.000	1.000
No	37	34		18	20		
Diabetes							
Yes	7	8	1.000	1	5	0.257	0.162
No	39	44		18	17		
Radscore, median (IQR)	− 1.6(− 2.6, − 0.5)	1.9 (0.9, 2.9)	< 0.001	− 0.6 (− 1.0, 0.2)	2.6 (1.2, 3.6)	< 0.001	0.583

*BMI* body mass index, *IQR* interquartile range.

^a^*p* value between the training and test cohorts.

### Outcomes performance of the clinical parameters model

The prediction model consisting of the clinical parameter for classifying the benign and malignant endometrial lesions returned the following performance metrics. The AUC was 0.70 (with a 95% CI 0.60–0.80) and the specificity, sensitivity, as well as accuracy were observed to be 54.3, 80.8, 68.4% respectively in the training cohort. For the test cohort, the AUC was 0.65 (with a 95% CI 0.49–0.81) and the specificity, sensitivity, and accuracy were found to be 57.9, 72.7, 65.9% respectively (see Table 2).

**Table 2:** Predictive performance of the radiomics nomogram, radiomics signature and the clinical models.

Cohort	Model	Accuracy (95% CI)	Sensitivity	Specificity
Training	Clinical	0.684 (0.582–0.774)	0.808	0.543
	Radiomics	0.857 (0.772–0.920)	0.942	0.761
	Nomogram	0.827(0.837–0.896)	0.750	0.913
Test	Clinical	0.659 (0.494–0.799)	0.727	0.579
	Radiomics	0.780 (0.624–0.894)	0.955	0.579
	Nomogram	0.805 (0.651–0.912)	0.850	0.762

*CI* confidence interval.

### Construction and assessment of the radiomics signature model

**Figure 2:**
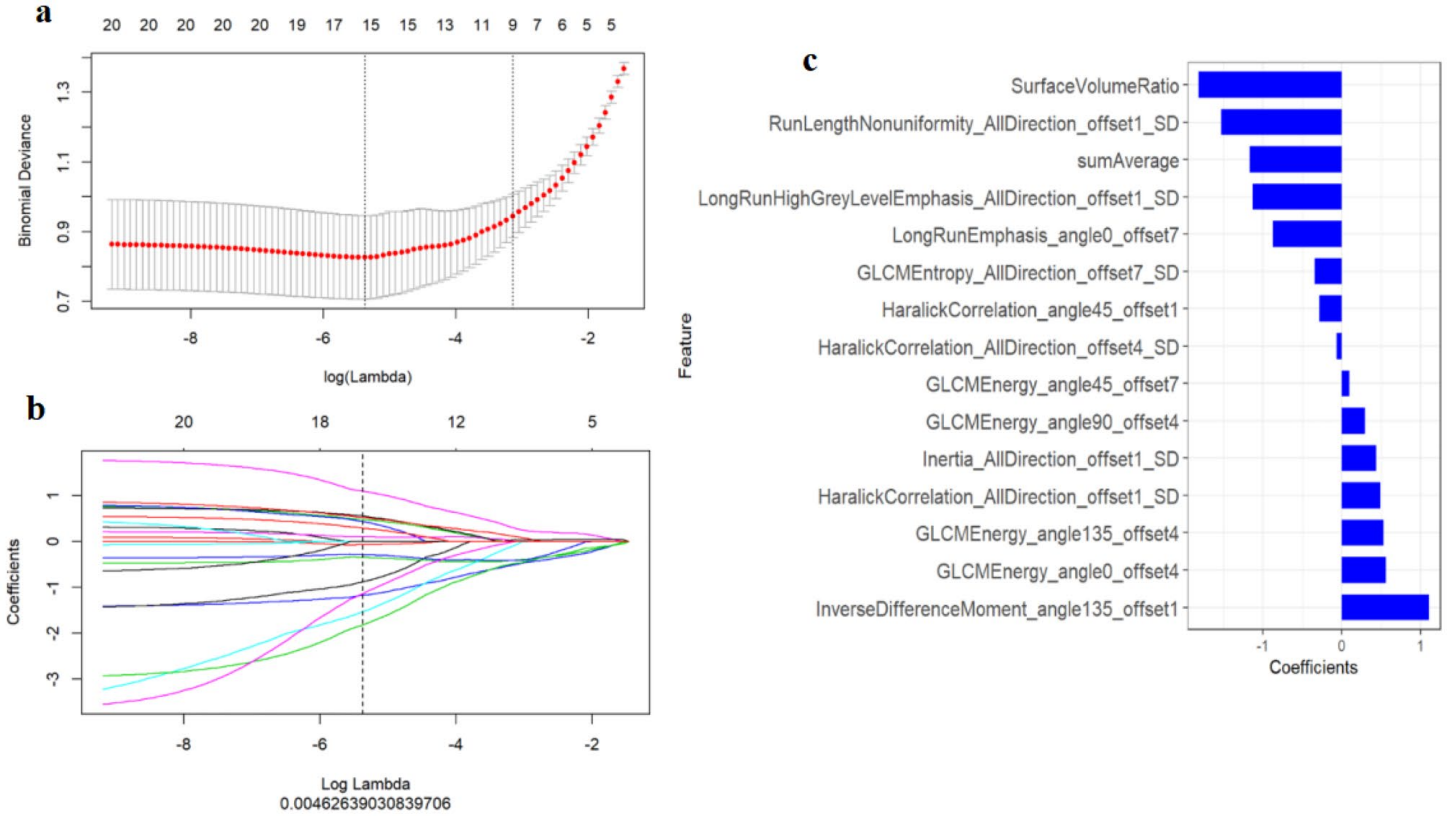
The selection of the various features and dimension reduction was performed using LASSO method. (**a**) Ten-fold cross-validation was used to choose the optimal parameter (λ) with the minimum criteria, thus determining the number of the features. (**b**) Coefficients for the optimal parameter (λ). A vertical line was drawn at the selected value of log (λ) and showed the coefficients with non-zero. (**c**) The final selected features and corresponding coefficients.

**Figure 3:**
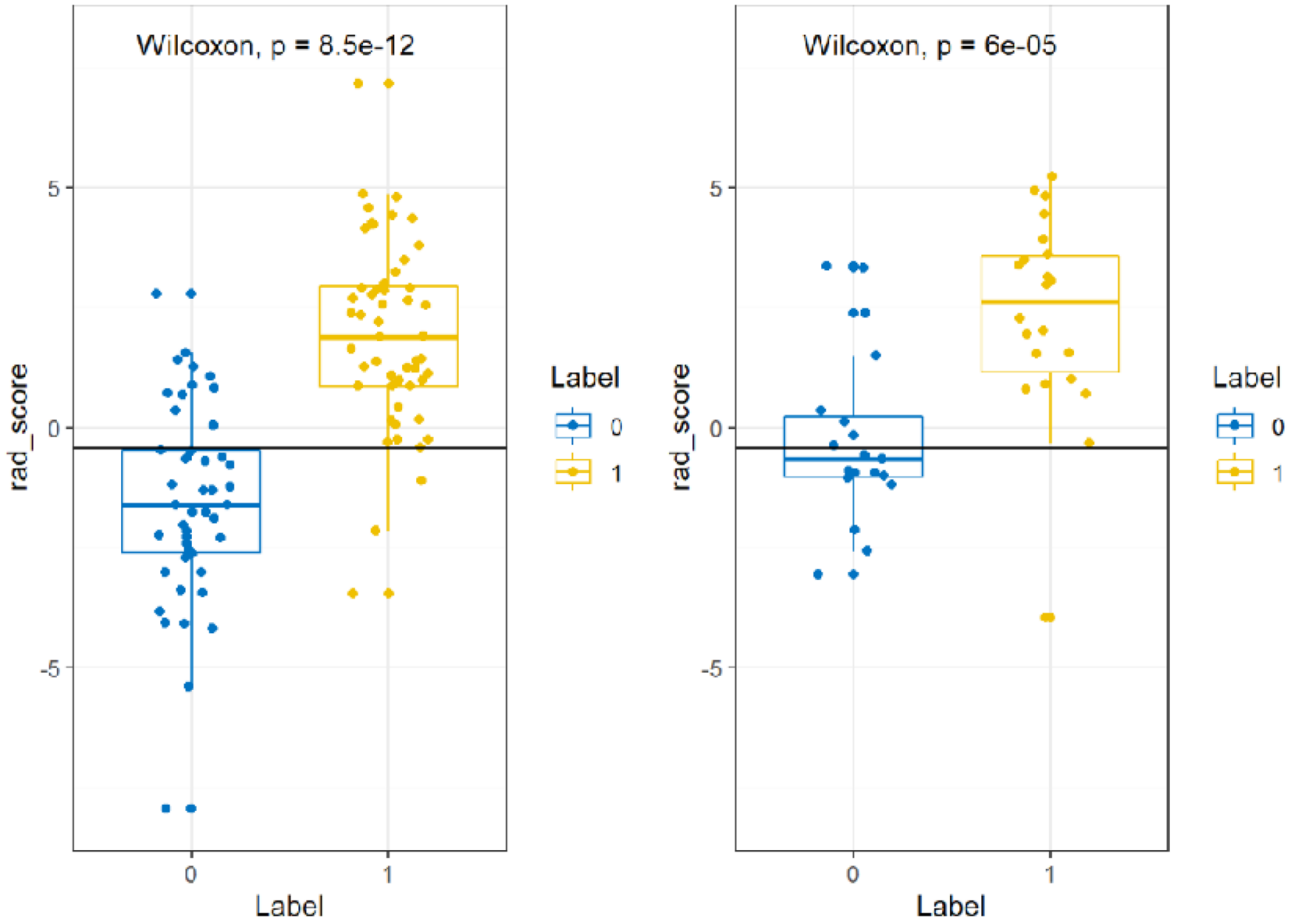
Radscore comparison of the benign and malignant endometrial lesions on the training and test cohort, respectively (left: training cohort; right: test cohort).

Features with ICC < 0.75 were removed, leaving 271/396 features (68.4%). After selection of the various features and dimension reduction, the top 15 important features were finally selected, and these were employed for the construction of radiomics signature model (see Fig. 2). The results indicated a good prediction performance for both the training and test data with only marginal differences. It was found that the radiomics signature model exhibited a satisfactory performance with AUC values of 0.90 (with a 95% CI 0.84–0.96) and 0.85 (with a 95% CI 0.72–0.98) in the training and test cohorts, specificity values of 76.1 and 57.9%, sensitivity values of 94.2 and 95.5%, and accuracy values of 85.7 and 78.0%, respectively (see Table 2). The radscore showed a significantly better discrimination both in the training and test data, comparing with clinical parameters model, thereby indicating the high potency of radiomics signature in the differential diagnosis of endometrial diseases (Fig. 3).

### Construction and assessment for the nomogram model

**Figure 4:**
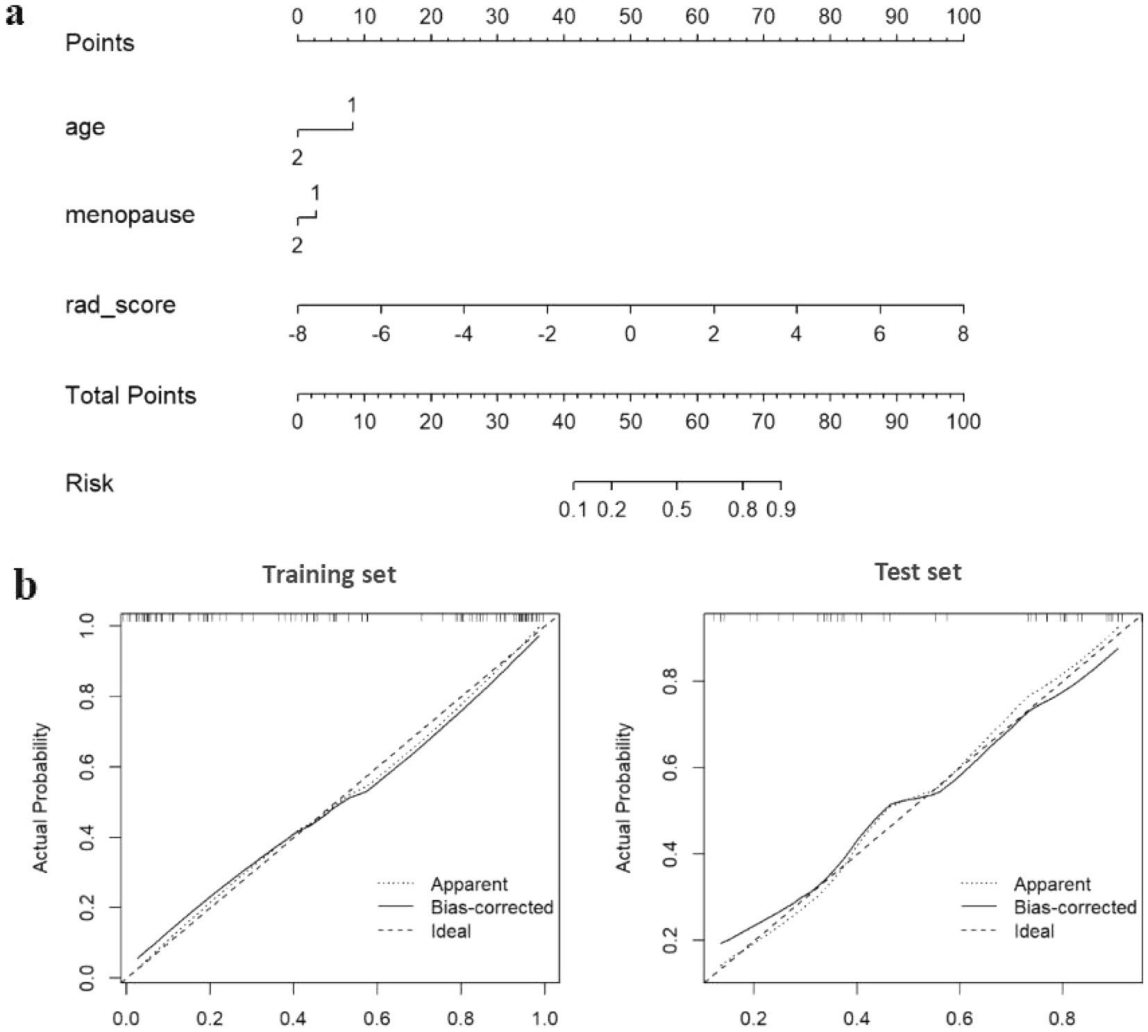
(**a**) A radiomics nomogram for determining the discrimination between benign and malignant endometrial lesions, which was developed in the training cohort. (**b**) The calibration curves of the training set (left) and the test set (right).

The age, menopause, hypertension were thereafter filtered as potentially clinical predictors of endometrial lesions by using a univariate logistic regression model (*p* < 0.1). For proper identification of benign and malignant endometrial diseases in patients, the most predictive subset of clinical parameters (age and menopause) was selected as they obtained the smallest value of Akaike information criterion (AIC) in the stepwise regression. Multivariate logistic regression finally yielded the three significant predictors including radscore, age, menopause, and lead to the construction of a more comprehensive and robust prediction model and nomogram (Fig. 4).

**Figure 5:**
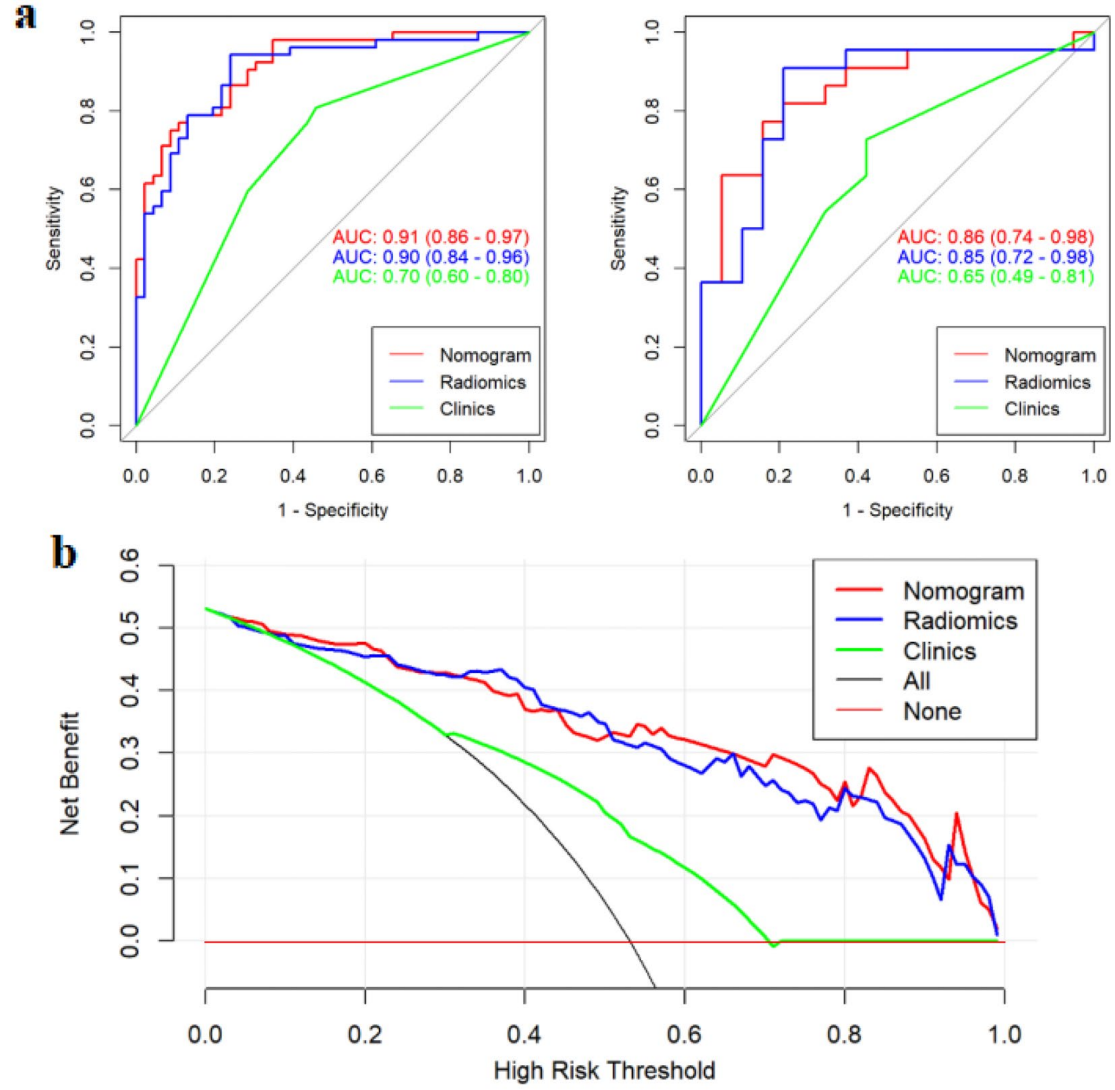
(**a**) The ROC curve and corresponding AUC values for the nomogram, radiomics signature and the clinical parameter models for identification of the benign and malignant endometrial lesions (left: training cohort; right: test cohort). (**b**) The decision curve analysis was used to evaluate the clinical benefit of the models. The green, blue and red lines correspond to the clinical parameter, radiomics signature and radiomics nomogram models respectively. The black line represents an extreme situation where all indicators are positive, while the horizontal red line represents the other extreme situation where all analyzed indicators are negative.

Radiomics nomogram showed excellent calibration potential in the prediction of properties of endometrial lesions through the calibration curves for both training and test cohorts (Fig. 4), but Hosmer–Lemeshow test indicated no statistical significance (*p* > 0.05). The AUCs of radiomics nomogram were 0.91 (with a 95% CI 0.86–0.97) and 0.86 (with a 95% CI 0.74–0.98) for the training and test sets respectively (Fig. 5). The specificity, sensitivity, accuracy were 91.3, 75.0, 82.7% for the training set, and 76.2%, 85.0%, 80.5% for the test set, respectively (Table 2).

Based on the results of DeLong test, the AUCs of the models were observed to be significantly different between the clinical indexes and nomogram in both the training and test cohorts (*p* < 0.05, see Table 3), which indicated a favorable prediction performance for the nomogram on both the cohorts. Moreover, according to the cut-off value of 0.566 for Youden index, the nomogram could potentially evaluate the risk score for endometrial lesions, and facilitate the categorization of patients into low- and high-risk groups. Figure 5 depicts the DCA plot of the radiomics nomogram. The outcomes indicated a more excellent risk prediction performance of the developed nomogram method as compared to the clinical parameters model.

**Table 3:** The prediction performances among the nomogram, radiomics signature and the clinical models.

Group	Model 1	Model 2	p value	Z value
Training	Clinical	Radiomics	0.001	3.477
	Radiomics	Nomogram	0.319	0.996
	Nomogram	Clinical	< 0.001	4.420
Test	Clinical	Radiomics	0.065	1.848
	Radiomics	Nomogram	0.832	0.212
	Nomogram	Clinical	0.016	2.400

p values and Z value were obtained by comparing the AUC value of ROC curve using Delong test.

## Discussion

The major outcome of this study was the construction of the novel classification model based on radiomics, which can effectively differentiate the patients with benign and malignant endometrial lesions. Both the nomogram and radiomics models show better efficiency than clinical parameters model, implying the clinical value of preoperative and non-invasive detection of EC. Although the nomogram and radiomics models exhibit comparable AUC value, nomogram could quantify the risk probability of endometrial lesions rather than unreadable high-dimensional features.

Robustness of the features extracted from images is essential for the development of the radiomics. Target segmentation, feature extraction, feature selection, and classification model implementation are affected by high inter- and intra-observer variability^23^. We chose manual segmentation because the dataset of this study is relatively small and ICC was calculated to improve the robustness and reproducible of the features. Radiologists can flexibly delineate targets manually resulting in highly accurate segmentation and manual segmentation is more intuitive and easily implemented way of obtaining a target lesion. Nevertheless, manual segmentation is labor-intensive, time-consuming, and not always feasible for radiomics analysis requiring huge datasets. The use of automatic or semi-automatic segmentation method could overcome the problem of strongly operator-dependent. Hence, most of radiomics studies with large datasets were based on automatic or semi-automatic segmentation methods. Many semi-automatic delineation algorithms, such as region growing or thresholding, are used in the clinical environment although less precise than manual segmentation^24^.

The study focused on FS T2WI images for radiomics data extraction, since there were informative demonstrations in previously reported radiomics studies^25,26^. Moreover, ADC images possess high requirement of magnetic field uniformity and have been found to be more susceptible to artifacts, especially in the pelvic region^13^. Dynamic contrast material-enhanced T1WI was not considered as the conventional sequence for some benign endometrial diseases. Herein, the 15 extracted features were comprised by the different parameters of Form Factor, Haralick, GLRLM and GLCM. The Form Factor features included detailed descriptors of the three-dimensional size and shape of the lesion region^27^, thus indicating the role of lesion size as an important predictor of the differential diagnosis for endometrial diseases^28^. GLCM features could also describe the complexity and level change in the values by quantifying the distribution of co-occurrence matrix^29^. Entropy and inertia of GLCM can reflect both the randomness of intensity image and the clarity of image respectively. Moreover, energy of GLCM and Haralick parameters emphasized the uniformity, known as local homogeneity^29^. CLRLM features primarily reflected roughness and directionality of the texture^30^.

The radiomics analysis has already been applied against endometrial disorders and exhibited superior outcomes, especially for EC. For example, Yan et al. constructed a radiomics model, which could precisely evaluate the conditions of pelvic lymph node, thus aiding with preoperative diagnosis of lymphatic metastasis of EC^31^. Moreover, another study developed innovative clinical-radiomics machine learning models and attempted to identify the various molecular anomalies of EC from contrast enhanced CT images noninvasively^32^. The major advantage of the nomogram is that the outcomes can provide the exactly risk stratification with general MRI image. The outcomes of nomogram contain an objective and specific probability of the correct scores of EC^9^, even if the model can’t be the ideal level of pathology and the misclassification is inevitable. It has been found that sometimes it can be difficult for the radiologists and gynecologists to give a specific judgment for their diagnoses preoperatively. The ability to utilize objective probability is of significant benefit for the diagnosis that could make up for misjudgment on part of the clinicians. Moreover, as the nomogram can provide definite information regarding these high-risk factors from MR images before treatment, it might help to effectively screen EC patients who might require more extensive surgery with preoperative risk stratification for optimal selection, and at the same time minimizing overtreatment of low risk patients.

There are few limitations associated with this study. First, the number of patients enrolled was relatively small, especially in case of patients with benign endometrial lesions which included multi-subtypes. Moreover, there were only five common types of endometrial lesions analyzed in the study, and cases with low incidence were not included. Second, the study did not perform the volumetric analysis, because it requires considerably more time and efforts. Hence, some important features connected with intrinsic heterogeneity might have been missed for the two-dimensional method. Lacking of multi-image study is another main limitation. Theoretically, every MRI sequences can be mined for huge information. Further validation utilizing prospective studies are also needed. It might also be essential to confirm the robustness and applicability for the prediction model, by carefully avoiding intrinsic biases and overfitting^33^. Hence, a larger and richer dataset will be needed to significantly strengthen our model and further validate the generalization ability, so that the model can be successfully applied to several different conditions.

## Conclusions

This study constructed a visualized nomogram model by combining radiomics signatures and the clinical parameters. The nomogram model exhibited an enormous clinical application value in predicting the objective probability of endometrial lesions classification non-invasive, and thus can aid the gynecologists for assessment and designing personalized treatment strategies. However, more efforts should be made to further improve applications and robustness of the model, so that it may become a real risk stratification tool for use in patients with endometrial lesions in the clinic.

## Data Availability

The data sets used and/or analyzed during the current study are available from the corresponding author on reasonable request.

## Abbreviations

ADC: Apparent dispersion coefficient
AIC: Akaike information criterion
AUC: Area under the receiver operation characteristic curve
BMI: Body mass index
CI: Confidence interval
CT: Computed tomography
DCA: Decision curve analysis
DWI: Diffusion weighted imaging
EC: Endometrial cancer
FOV: Field of view
FS-T2WI: T2-weighted image with fat saturation
GLCM: Gray level co-occurrence matrix
GLRLM: Gray level run-length matrix
GLZSM: Gray level zone-size matrix
ICC: Intra-class correlation coefficient
LASSO: The least absolute shrinkage and selection operator
MRI: Magnetic resonance imaging
mRMR: Maximum relevance minimum redundancy
ROC: Receiver operation characteristic
ROI: Region of interest
TE: Echo time
TR: Repetition time
T1WI: T1-weighted image
T2WI: T2-weighted image
